# Low levels of small HDL particles predict but do not influence risk of sepsis

**DOI:** 10.1186/s13054-023-04589-1

**Published:** 2023-10-09

**Authors:** Fergus Hamilton, Kasper Mønsted Pedersen, Peter Ghazal, Børge Grønne Nordestgaard, George Davey Smith

**Affiliations:** 1 MRC Integrative Epidemiology Unit, University of Bristol, Oakfield House, Oakfield Road, Bristol, BS8 2PS UK; 2 Infection Science, North Bristol NHS Trust, Bristol, UK; 3 Department of Clinical Biochemistry, Herlev and Gentofte Hospital, Copenhagen University Hospital, Herlev, Denmark; 4 Institute of Clinical Medicine, Faculty of Health and Medical Sciences, University of Copenhagen, Copenhagen, Denmark; 5 Project Sepsis, Cardiff University, Cardiff, UK

## Abstract

**Background:**

Low levels of high-density lipoprotein (HDL) cholesterol have been associated with higher rates and severity of infection. Alterations in inflammatory mediators and infection are associated with alterations in HDL cholesterol. It is unknown whether the association between HDL and infection is present for all particle sizes, and whether the observed associations are confounded by IL-6 signalling.

**Methods:**

In the UK Biobank, ~ 270,000 individuals have data on HDL subclasses derived from nuclear magnetic resonance analysis. We estimated the association of particle count of total HDL and HDL subclasses (small, medium, large, and extra-large HDL) with sepsis, sepsis-related death, and critical care admission in a Cox regression model. We subsequently utilised genetic data from UK Biobank and FinnGen to perform Mendelian randomisation (MR) of each HDL subclass and sepsis to test for a causal relationship. Finally, we explored the role of IL-6 signalling as a potential causal driver of changes in HDL subclasses.

**Results:**

In observational analyses, higher particle count of small HDL was associated with protection from sepsis (Hazard ratio, HR 0.80; 95% CI 0.74–0.86, *p* = 4 × 10^–9^ comparing Quartile 4, highest quartile of HDL to Quartile 1, lowest quartile of HDL), sepsis-related death (HR 0.80; 95% CI 0.74–0.86, *p* = 2 × 10^–4^), and critical care admission with sepsis (HR 0.72 95% CI 0.60–0.85, *p* = 2 × 10^–4^). Parallel associations with other HDL subclasses were likely driven by changes in the small HDL compartment. MR analyses did not strongly support causality of small HDL particle count on sepsis incidence (Odds ratio, OR 0.98; 95% CI 0.89–1.07, *p* = 0.6) or death (OR 0.94, 95% CI 0.75–1.17, *p* = 0.56), although the estimate on critical care admission with sepsis supported protection (OR 0.73, 95% CI 0.57–0.95, *p* = 0.02). Bidirectional MR analyses suggested that increased IL-6 signalling was associated with reductions in both small (beta on small HDL particle count − 0.16, 95% CI − 0.10 to − 0.21 per natural log change in SD-scaled CRP, *p* = 9 × 10^–8^).and total HDL particle count (beta − 0.13, 95% CI − 0.09 to − 0.17, *p* = 7 × 10^–10^), but that the reverse effect of HDL on IL-6 signalling was largely null.

**Conclusions:**

Low number of small HDL particles are associated with increased hazard of sepsis, sepsis-related death, and sepsis-related critical care admission. However, genetic analyses did not strongly support this as causal. Instead, we demonstrate that increased IL-6 signalling, which is known to alter infection risk, could confound associations with reduced HDL particle count, and suggest this may explain part of the observed association between (small) HDL particle count and sepsis.

**Supplementary Information:**

The online version contains supplementary material available at 10.1186/s13054-023-04589-1.

## Introduction

Lipid and lipoprotein pathways are increasingly recognised as an integral part of immunity and infection [1]. These connections studied in animal models include bidirectional mechanistic links in immunometabolism, of which functional alterations in the metabolic development of high-density lipoprotein (HDL) by the acute phase response are important [1–3]. In humans, multiple observational studies have identified changes in lipids and lipoproteins in patients with sepsis [2], while population-based epidemiological studies have identified associations between certain lipid and lipoprotein classes and the risk of severe infection [2, 4–6].

The most compelling data sit with HDL [3, 7, 8]. This lipoprotein class has a long history in epidemiological research, as original observations finding an inverse association of HDL cholesterol concentrations in blood and cardiovascular disease (earning it the unfortunate moniker “good cholesterol”), with subsequent randomised trial and genetic evidence compellingly suggesting no fundamental role in circulating HDL cholesterol in atherothrombotic cardiovascular disease [3, 9, 10].

However, in the acute phase response HDL particle concentration, function, and structure change [2]. Multiple lines of evidence point to a potential biological role of these altered HDL particles in infection and inflammation. Firstly, HDL is conserved across many biological species despite no clear biological role; however, HDL has been described to be involved in bacterial lipopolysaccharide binding, clot formation, and wound healing [3, 4, 11]. Secondly, multiple studies have identified rapid reductions in HDL cholesterol (tracking HDL particle count) and other changes in HDL particle character during sepsis episodes, with the extent of reductions being linked to increased mortality [2, 7, 12–14]. Thirdly, a single placebo-controlled randomised trial of extended release niacin unexpectedly showed an increase in infection in those assigned to niacin, which was also shown to increase HDL cholesterol [15]. Fourthly, experimental data support HDL administration being protective in animal models of sepsis, with human trials to alter lipid biology in sepsis ongoing [16–19]. Finally, a recent study using single-nucleotide polymorphisms (SNPs) that are associated with HDL levels found that genetically predicted higher HDL concentration was associated with lower odds of sepsis [20]. In more targeted analysis, SNPs in *CETP* that lead to higher HDL cholesterol levels appear to decrease the odds of sepsis [21]. However, placebo-controlled randomised trials of CETP inhibitors did not show clear differences in infection rates between arms, despite increases in HDL in the treatment arm [22–24]; that said, these studies were not powered at or aimed to examine risk infection.

Recently, nuclear magnetic resonance (NMR) spectroscopy has allowed more detailed exploration of lipoprotein subclasses in large population studies [25–27]. With these techniques, reliable measures of HDL subclasses (defined by particle size and lipid content) have become available [28]. This finer resolution is critical for understanding the underlying biology and whether all or just particular types or particle sizes of HDL are relevant for sepsis, which can then be used to prioritise further analyses (e.g. randomised trials and genetic analyses).

Recent NMR-based lipoprotein data from the Copenhagen General Population Study were used to identify protective association with sepsis with increased numbers of small and medium sized HDL particles, with no association identified with larger HDL particles [29]. However, this finding has not yet been replicated. In the present study in UK Biobank, a large, prospective collected volunteer cohort aimed to test the association between lipoprotein particle count of specific HDL subclasses and severe infection (sepsis), using both an observational approach (NMR-based HDL subclass data, *n* ~ 270,000) and a genetic approach (Mendelian randomisation [MR], *n* ~ 450,000).

Subsequently, we examined whether IL-6 signalling could confound observational estimates between HDL subclass particle counts and infection [30, 31]. We chose to focus on IL-6 signalling as the bidirectional link between inflammation and cholesterol metabolism is well established, and IL-6 is a key and highly pleiotropic cytokine in the innate immune response. Additionally, reduced IL-6 signalling in both randomised trials and genetic studies) alters both HDL levels [32–34] and infection risk [30, 35, 36].

## Methods

**Fig. 1:**
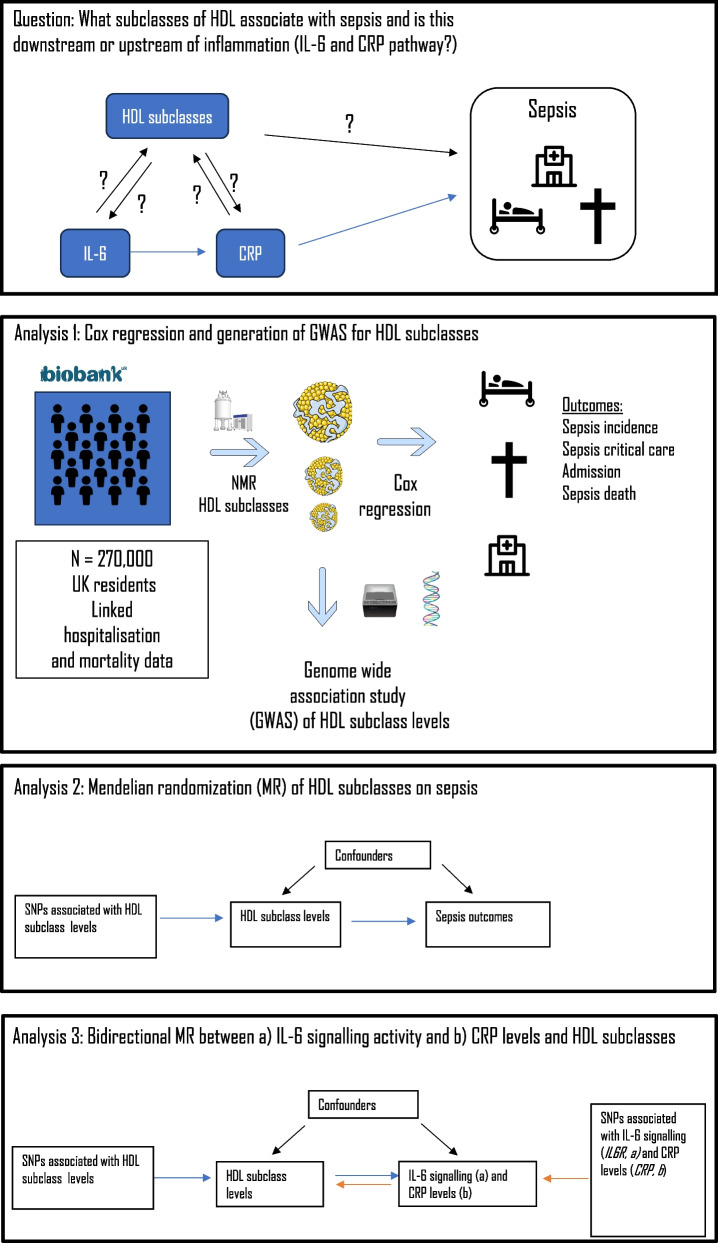
An overview of the overarching question and the three analyses performed

We provide an overview of our analytic approach in Fig. 1.

### Data sources

This analysis was performed in UK Biobank, a large volunteer cohort of around 500,000 individuals [37]. Participants were recruited between 2006 and 2012 across 21 UK sites and had blood samples taken on recruitment [37]. Participants were then followed up using linked, national electronic health records for subsequent healthcare events (e.g. hospitalisations), and likewise linked to national mortality data. For this study, the observational analyses were performed on the random subset of the cohort that had NMR spectroscopy performed on the baseline plasma sample (*n* ~ 270,000), and genetic analyses were conducted on those of European ancestry (defined below) with linked genetic data (*n* ~ 450,000). For specific analyses on critical care admission, the sample was limited to those recruited in England (approximately 70% of the sample size), as linked critical care data are only available in England.

Around 20,000 UK Biobank participants were invited back for a repeat assessment centre visit around 5 years after the initial assessment (between 2012 and 2013). Around 17,000 of these had NMR-based lipoprotein data from samples collected at this time.

Additional sepsis outcomes were recorded in FinnGen, a contemporary Finnish Biobank that includes around 330,000 Finnish residents with linked electronic health record data [38].

### NMR lipoprotein data

Lipoprotein subclass data were measured using an established NMR platform developed by Nightingale Health Plc (Helsinki, Finland) [28, 39, 40]. The measurements took place between 2019 and 2022 using eight spectrometers at Nightingale Health Plc. For the present analysis, we focussed on the particle count of HDL subclasses and total HDL. In line with recent literature and the Nightingale Health NMR platform description, we categorised particles as small HDL particles (< 9.8 nm), medium HDL particles (9.8–11.5 nm), large HDL particles (11.6–13.2 nm), and extra-large HDL particles (13.3–16.5 nm) [29, 39, 41]. For this analysis, we primarily focused on the 270,000 initial samples taken on recruitment to UK Biobank, although we performed a secondary analysis on the repeat samples, in which around 15,000 participants had initial and repeat HDL measures. Fifty-seven separate HDL cholesterol measurements were measured across four sizes (S, M, L, XL), alongside global measurements (e.g. total HDL). Complete data were available for nearly all participants, with less than 1% of results missing across each HDL measure. Additional file 1: Table S1 reports the measurements in more detail.

### Definitions of outcomes and covariates

Sepsis outcomes were defined using hospital coding in line with recent work [30]. We extracted all ICD-10 codes for sepsis (A39, A40, A41) from linked hospitalisation (HES) data in England (and similar datasets in the devolved nations) from after recruitment to UK Biobank [37].

Linked critical care data were available for all participants in England. Critical care admission was defined as any admission to a critical care unit (for level 2 or level 3 care) during the index admission for sepsis. All-cause mortality was extracted from linked national mortality data, and deaths from sepsis were considered as any death within 28 days of an admission with sepsis.

We extracted data on relevant covariates from UK Biobank, from samples or examinations taken on recruitment. We extracted data on renal function (serum creatinine), body mass index, inflammation (C-reactive protein), age, sex, UK Biobank recruitment centre, diabetes, liver disease, cancer, smoking, alcohol history, and usage of statins. We extracted an individual measure of socio-economic deprivation on recruitment, the Townsend deprivation index [42]. As the sample was taken at recruitment, we utilised self-reported statin usage on recruitment (the same day as sampling) to define statin users. Statin codes were identified from a recent publication [43]. Follow-up was performed until March 2021.

### Observational analysis (analysis 1)

Observational analyses were focussed on the association between measured particle count of HDL subclasses on recruitment to UK Biobank and the risk of (a) hospitalisation with sepsis; (b) critical care admission with sepsis, and (c) death within 28 days of sepsis. Our primary statistical analysis was the association between particle count of HDL subclasses and the incidence of sepsis using time to event analyses, using Cox regression. For the linear models, given the large differences in absolute particle counts, we scaled each particle to have a mean of zero and standard deviation (SD) of 1, so estimates should be interpreted per one SD change in the exposure. Nonlinearity was explored using restricted cubic spline models and via splitting the exposure into quartiles. Analyses were adjusted for age, sex, body mass index, C-reactive protein level, renal function, history of diabetes, history of liver disease, history of cancer, a composite measure of socio-economic state (Townsend deprivation index), statin usage, and smoking and alcohol usage. These were included as potential confounders or proxies for unmeasured confounders.

Missing data were imputed using multiple imputation via the *mice* package in R, and we report our primary analyses on the imputed data, although results without imputation were similar. Analyses were conducted in R 4.0.4 using the packages *survival, rms* and *mice* [44–46].

### Sensitivity and secondary analyses

IL-6 is a cytokine known to regulate CRP expression levels, a commonly measured acute phase reactant [47]. Higher levels of IL-6 have been associated with lower levels of HDL [48] and specifically small HDL [49]. Further, reducing IL-6 signalling is associated with a moderately increased risk of infection in randomised trials of IL-6 antagonists [35], although this association is complicated by the apparent benefits of reduced IL-6 activity in (some) severe infection [30, 36, 50–52]. IL-6 has two major forms of signalling, “classical” signalling, which is mediated by IL-6 binding to a membrane bound receptor (IL6R), and “*trans*” signalling, where IL-6 binds to the soluble form of the IL-6 receptor. The relative contribution of these to disease states is still an unresolved question, with drugs available that target all forms of IL-6 signalling (e.g. IL-6 receptor antagonists), and specific receptors for trans signalling [53]. We test the role of IL-6 signalling on HDL measures extensively in our subsequent genetic analysis (Analysis 3), but we also tested whether associations with HDL subclasses observationally changed in models without adjustment for CRP, as the best available measure of IL-6 signalling in this dataset.

Statin usage is known to substantially alter lipid values although this is largely related to lipoprotein classes outside HDL [54]. However to identify if there was evidence of any effect modification of the effect of HDL lipoprotein particle counts on sepsis with statin usage, we performed analyses in all participants (with statins usage as a covariate) and performed sensitivity analyses stratified by statin use.

### Genetic analyses (Analyses 2 and 3)

To perform genetic analysis, we used Mendelian randomisation (MR) [55]. This is an approach whereby genetic variants that are robustly associated with the exposure of interest (e.g. small HDL levels) are used as instrumental variables to attempt to identify a causal estimate of the effect of this exposure on an outcome (e.g. sepsis). There are three fundamental assumptions of MR that are required for a causal interpretation: (1) The variant(s) are associated with the exposure (relevance); (2) There are no confounders of the variant and the outcome (3) The variant (s) do not affect the outcome other than through the exposure (exclusion restriction).This approach has been widely used in the genetic epidemiology of lipoproteins, with MR analysis on HDL levels consistently identifying no causal effect on cardiovascular outcomes, a finding also identified in multiple randomised trials [9, 56]. Our specific analytic approach is described below.

### Analysis 2: Is small and/or total HDL causally associated with sepsis-related outcomes?

Firstly, we aimed to test whether HDL or small HDL was causally associated with sepsis and sepsis-related mortality and critical care admission. We therefore performed two-sample MR using genetic variants associated with small HDL and total HDL particle number as instruments in an instrumental variable analysis [55, 57]. To identify variants for HDL subclasses, we generated GWAS for total HDL particle count and each HDL subclass measure in UK Biobank using quality-controlled genetic data. Details on GWAS methodology are available in the Additional file 2.

Subsequently, we measured sepsis outcomes by utilising our previously performed GWAS of sepsis in UK Biobank [30], with additional sepsis outcomes replicated in FinnGen, a large prospective cohort in Finland [58]. In both of these studies, ICD-10 coding was used to define sepsis cases (UK Biobank; A39, A40, A41; FinnGen: A40, 41). In UK Biobank, we also utilised GWAS for sepsis-related critical care admission, and GWAS for sepsis-related mortality. Both of these were derived from nationally linked electronic data records. Details of GWAS methodology for the outcome data are available with the relevant publications. [58, 59]

For each exposure GWAS, instruments were identified identically. Variants that were associated with the exposure (*p* < 5 × 10^–8^) and independent (*R*^2^ =  < 0.001 in European ancestry participants of the 1000 Genomes Project) were identified and taken forward. Effect estimates for each SNP were then extracted and harmonised from the outcome dataset. Where SNPs were not available, LD proxies were identified (min *R*^2^ 0.8 in 1000 Genomes Project, European ancestry participants).

Our primary analytical method was to combine all SNP-exposure and SNP-outcome associations with fixed effect inverse variance weighting (IVW-MR), but we also report MR Egger, weighted median and MR-PRESSO, all approaches that aim to alleviate the effect of horizontal pleiotropy on estimates [60, 61]. Analyses were performed using the TwoSampleMR package in R 4.0.4 [62].

Additionally, to explore the effect of the small HDL genetic associations on phenotype and to visualised pleiotropy, we generated a polygenic risk score (PRS) for each participant using the same genetic variants used in our two-sample MR. This was fitted using PLINK 2.0.4 [63].

### Analysis 3: Is IL-6 signalling activity a potential risk factor for HDL particle counts?

To further explore IL-6 signalling and the effect on HDL particle counts, we performed bidirectional MR. Firstly, we utilised variants cis to *IL6R* as instruments to proxy IL-6 signalling and to explore the effect of IL-6 signalling on HDL and HDL subclasses. Using variants *cis* to *IL6R* to proxy IL-6 signalling is a common MR approach, and is used widely and is recognised to phenocopy IL-6 receptor inhibition with drugs such as IL-6 receptor antagonists [30, 34, 36, 64].

We used 26 variants within 300 kb of IL6R and all associated (at genome wide significance, *p* < 5 × 10^–8^) with CRP. These variants come from a recent meta-analysis between UK Biobank and CHARGE [65]. CRP is a widely used read-out of classical IL-6 activity and reflects the effect of *cis* IL-6 signalling on hepatocytes [53]. However, it is important to recognise that although we describe this as “reduced IL-6 signalling”, and the genetic literature supports similar effects to IL-6 blockade in trials, the effect on *trans* signalling with these genetic variants remains incompletely understood at present. We then performed a complementary analysis of the effect of CRP on HDL subclasses using 4 well-characterised variants cis to *CRP*, from our recent study [30], to see whether any effect was driven by changes in CRP itself.

Subsequently, we then performed MR of the effect of HDL subclasses on IL-6 and CRP levels, i.e. in the “reverse” direction. This approach allows us to identify if the causal effect is in the other direction, i.e. changes in HDL levels lead to changes in CRP and/or IL-6. We used the same approach to identifying variants associated with HDL subclasses as in our analysis on sepsis outcomes. Our outcome GWAS for CRP was from a recent meta-analysis (*n* ~ 500,000) [65], while our outcome GWAS for IL-6 comes from the European ancestry subset of UK Biobank Pharma Proteomic Project (*n* ~ 37,000) [66]. We performed two-sample MR and applied a Steiger filter [67]. This filters all variants that explain more of the variance in the outcome than the exposure, and can provide reassurance that the variants are in the appropriate causal direction.

### Statin usage and its effect on lipoprotein subclasses

As statin usage is known to distort genotype–metabolite relationships [54, 68], we performed additional analyses to explore whether the association between small HDL measures and genotype was altered and how this could affect results. For this, we performed a GWAS in statin users (defined as per the observational analyses), and non-statin users, and calculated the genetic correlation using LD score regression [69]. As this approach is likely to lead to a collider bias due to the association between statin use and cholesterol measures, we also performed a GWAS of small HDL in those under 50, where statin use was expected to be rare (n = 994/23,532, 4%) and we would avoid the collider bias issue (at the expense of statistical power), and assessed the genetic correlation between this GWAS and the GWAS in statin users and non-users.

### Sample overlap and its effect on estimates

Although summary statistics from GWAS of both sepsis [24] and HDL subclass measures [70] are available already, the largest samples (by an order of magnitude) are in UK Biobank, leading to sample overlap in the exposure and outcome datasets, which leads to “winners curse”, biasing estimates [55, 71]. In order to maximise sample size while reducing overfitting, we performed a sensitivity analysis using block jacknife resampling [72]. We split UK Biobank into multiple blocks using a block jacknife approach. Firstly, we performed a GWAS on all ~ 260,000 participants with small HDL particle count numbers and genetic data on recruitment to UK Biobank and used this to generate instruments for small HDL. We then measured outcomes (by performing a GWAS for each outcome) in the ~ 210,000 participants who had no small HDL measures. As there is no sample overlap between these groups, this represents traditional two-sample MR.

To measure outcomes in the remaining ~ 260,000 participants, we used a block resampling approach [72]. To do this we split this group into ten further samples, each with around 26,000 participants. We measured the outcome in each block independently and used a GWAS for the exposure performed in the other blocks to generate instruments without sample overlap. For example, we combined groups 1–9 and performed a GWAS of small HDL measures (*n* ~ 234,000). We then generated instruments (as described above) in this block and used these instruments on outcomes we had measured in block 10 (*n* ~ 26,000). We then repeated this for all other blocks. In this way, ten estimates are generated (one for each block). These estimates were then combined, along with the estimate in the 210,000 UK Biobank participants without small HDL measures, to generate a summary MR estimate, using fixed effects meta-analysis. This approach has been shown to effectively reduce bias from sample overlap [72]. As these results were similar to the main analyses, these are presented entirely in a supplement (Additional file 1).

### Reporting guidelines

This study was performed in line with the STROBE-MR reporting guidelines. A checklist is attached as an Additional file 3.

## Results

### Demographics and study cohort

Our observational dataset with NMR measures included 259,908 participants, of an average age of 59 on recruitment, with 54% of the cohort being female. Details on demographics, age, sex, and comorbidities are presented in Table 1, stratified by quartiles of small HDL cholesterol. As NMR measures were performed at random on the cohort, there were limited differences between the full UKB cohort and the sampled cohort (Additional file 1: Table S2). There were differences between small HDL quartiles across both demographic and clinical strata. The most marked difference was for sex (48% female lowest quartile, 60% highest quartile). Across the cohort, there were 5961 cases of sepsis requiring hospital admission, occurring a median of 2956 days (~ 8 years, IQR 1980–3536) days after study entry. The median total follow-up time was 4089 days (~ 11.2 years), leading to a total study period of 5,167,580 person-years. A total of 1118 participants died within 28 days of a sepsis admission, a 28-day mortality of 15.7%. Most participants resided in England (*n* = 234,983), where data on critical care admission were available. Of those admitted to hospital in England with sepsis, 769 out of 5859 (13%) participants had a critical care admission in their index hospital admission for sepsis.

**Table 1:** Demographics of the observational cohort, stratified by number of small HDL particles

Characteristic	Quartile 1, *N* = 64,752^a^	Quartile 2, *N* = 64,752^a^	Quartile 3, *N* = 64,752^a^	Quartile 4, *N* = 64,752^a^	*p* value^b^
Age on recruitment	59 (50, 64)	59 (51, 64)	59 (51, 64)	59 (52, 64)	< 0.001
Sex					< 0.001
Female	31,126 (48%)	33,606 (52%)	36,008 (56%)	39,152 (60%)	
Male	33,626 (52%)	31,146 (48%)	28,744 (44%)	25,600 (40%)	
Body mass index (kg/m^2^)	26.4 (23.7, 29.8)	26.7 (24.1, 29.9)	26.8 (24.3, 29.9)	27.0 (24.5, 30.0)	< 0.001
Diabetes					< 0.001
No	59,672 (92%)	61,399 (95%)	62,043 (96%)	62,421 (96%)	
Yes	4,849 (7.5%)	3,185 (4.9%)	2,557 (4.0%)	2,157 (3.3%)	
Do not know	174 (0.3%)	117 (0.2%)	111 (0.2%)	135 (0.2%)	
Unknown	37	29	25	20	
History of liver disease	2,147 (3.3%)	1,620 (2.5%)	1,594 (2.5%)	1,651 (2.5%)	< 0.001
History of cancer	5,986 (9.2%)	5,914 (9.1%)	6,039 (9.3%)	6,151 (9.5%)	0.14
C-reactive protein level (mg/dl)	1.32 (0.61, 3.01)	1.30 (0.64, 2.68)	1.30 (0.66, 2.63)	1.40 (0.72, 2.74)	< 0.001
Unknown	3,269	2,844	2,786	2,756	
Creatinine (umol/L)	72 (63, 83)	71 (62, 82)	70 (61, 80)	68 (60, 78)	< 0.001
Unknown	3,082	2,754	2,716	2,701	
Statin user	13,357 (21%)	11,703 (18%)	11,188 (17%)	11,556 (18%)	< 0.001
Alcohol intake frequency					< 0.001
Daily or almost daily	9,173 (14%)	11,379 (18%)	13,928 (22%)	19,183 (30%)	
Never	6,126 (9.5%)	4,742 (7.3%)	3,766 (5.8%)	2,912 (4.5%)	
Once or twice a week	18,175 (28%)	18,191 (28%)	17,331 (27%)	14,955 (23%)	
One to three times a month	9,112 (14%)	7,932 (12%)	6,784 (10%)	5,150 (8.0%)	
Prefer not to answer	65 (0.1%)	54 (< 0.1%)	38 (< 0.1%)	58 (< 0.1%)	
Special occasions only	9,083 (14%)	7,459 (12%)	6,459 (10.0%)	5,026 (7.8%)	
Three or four times a week	12,981 (20%)	14,967 (23%)	16,422 (25%)	17,448 (27%)	
Unknown	37	28	24	20	
Smoking status					< 0.001
Current	7,503 (12%)	6,762 (10%)	6,377 (9.9%)	6,477 (10%)	
Never	35,589 (55%)	35,806 (55%)	35,000 (54%)	32,541 (50%)	
Prefer not to answer	235 (0.4%)	231 (0.4%)	213 (0.3%)	273 (0.4%)	
Previous	21,388 (33%)	21,925 (34%)	23,138 (36%)	25,442 (39%)	
Unknown	37	28	24	19	
Townsend deprivation index (lower is more deprived)	− 2.25 (− 3.69, 0.36)	− 2.32 (− 3.74, 0.18)	− 2.34 (− 3.73, 0.11)	− 2.28 (− 3.70, 0.21)	< 0.001
Unknown	78	75	68	77	
Total cholesterol (mmol/L)	3.94 (3.41, 4.48)	4.45 (3.96, 4.95)	4.80 (4.29, 5.31)	5.31 (4.76, 5.89)	< 0.001
HDL cholesterol (mmol/L)	1.09 (0.92, 1.35)	1.20 (1.05, 1.43)	1.30 (1.15, 1.52)	1.47 (1.30, 1.70)	< 0.001
LDL cholesterol (mmol/L)	1.42 (1.19, 1.67)	1.66 (1.43, 1.91)	1.82 (1.58, 2.08)	2.04 (1.77, 2.33)	< 0.001
Total triglycerides (mmol/L)	1.08 (0.79, 1.48)	1.19 (0.88, 1.62)	1.25 (0.92, 1.69)	1.33 (0.98, 1.80)	< 0.001
Sepsis	1,921 (3.0%)	1,440 (2.2%)	1,347 (2.1%)	1,253 (1.9%)	< 0.001
Sepsis death	387 (0.6%)	262 (0.4%)	259 (0.4%)	210 (0.3%)	< 0.001
Sepsis critical care admission	273 (0.4%)	183 (0.3%)	155 (0.2%)	162 (0.3%)	< 0.001

^a^Median (IQR); *n* (%); Range

^b^Kruskal–Wallis rank sum test; Pearson's Chi-squared test

**Fig. 2:**
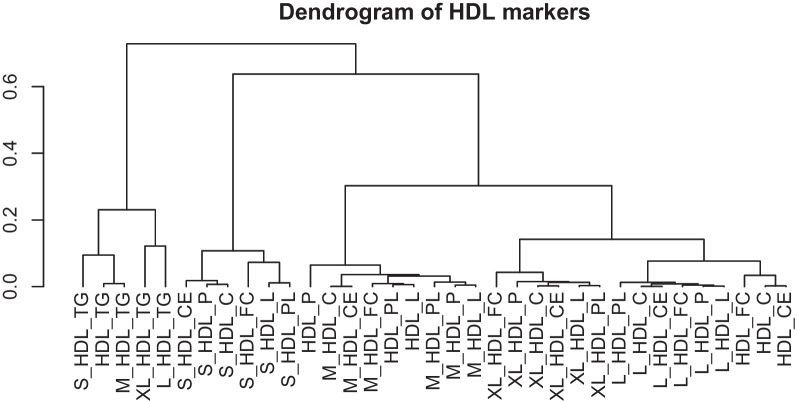
Dendrogram of correlation across all HDL measures in UK Biobank. Measures are grouped by their correlation, with branches lower showing increasing correlation. Abbreviations: HDL_C = HDL Cholesterol, HDL_TG = Triglycerides in HDL, HDL_PL = Phospholipids in HDL, HDL_CE = Cholesteryl Esters in HDL, HDL_FC = Free Cholesterol in HDL, HDL_L = Total Lipids in HDL, HDL_P = Concentration of HDL Particles, XL_HDL_P = Concentration of Very Large HDL Particles, XL_HDL_L = Total Lipids in Very Large HDL, XL_HDL_PL = Phospholipids in Very Large HDL, XL_HDL_C = Cholesterol in Very Large HDL, XL_HDL_CE = Cholesteryl Esters in Very Large HDL, XL_HDL_FC = Free Cholesterol in Very Large HDL, XL_HDL_TG = Triglycerides in Very Large HDL, L_HDL_P = Concentration of Large HDL Particles, L_HDL_L = Total Lipids in Large HDL, L_HDL_PL = Phospholipids in Large HDL, L_HDL_C = Cholesterol in Large HDL, L_HDL_CE = Cholesteryl Esters in Large HDL, L_HDL_FC = Free Cholesterol in Large HDL, L_HDL_TG = Triglycerides in Large HDL, M_HDL_P = Concentration of Medium HDL Particles, M_HDL_L = Total Lipids in Medium HDL, M_HDL_PL = Phospholipids in Medium HDL, M_HDL_C = Cholesterol in Medium HDL, M_HDL_CE = Cholesteryl Esters in Medium HDL, M_HDL_FC = Free Cholesterol in Medium HDL, M_HDL_TG = Triglycerides in Medium HDL, S_HDL_P = Concentration of Small HDL Particles, S_HDL_L = Total Lipids in Small HDL, S_HDL_PL = Phospholipids in Small HDL, S_HDL_C = Cholesterol in Small HDL, S_HDL_CE = Cholesteryl Esters in Small HDL, S_HDL_FC = Free Cholesterol in Small HDL, S_HDL_TG = Triglycerides in Small HDL

**Fig. 3:**
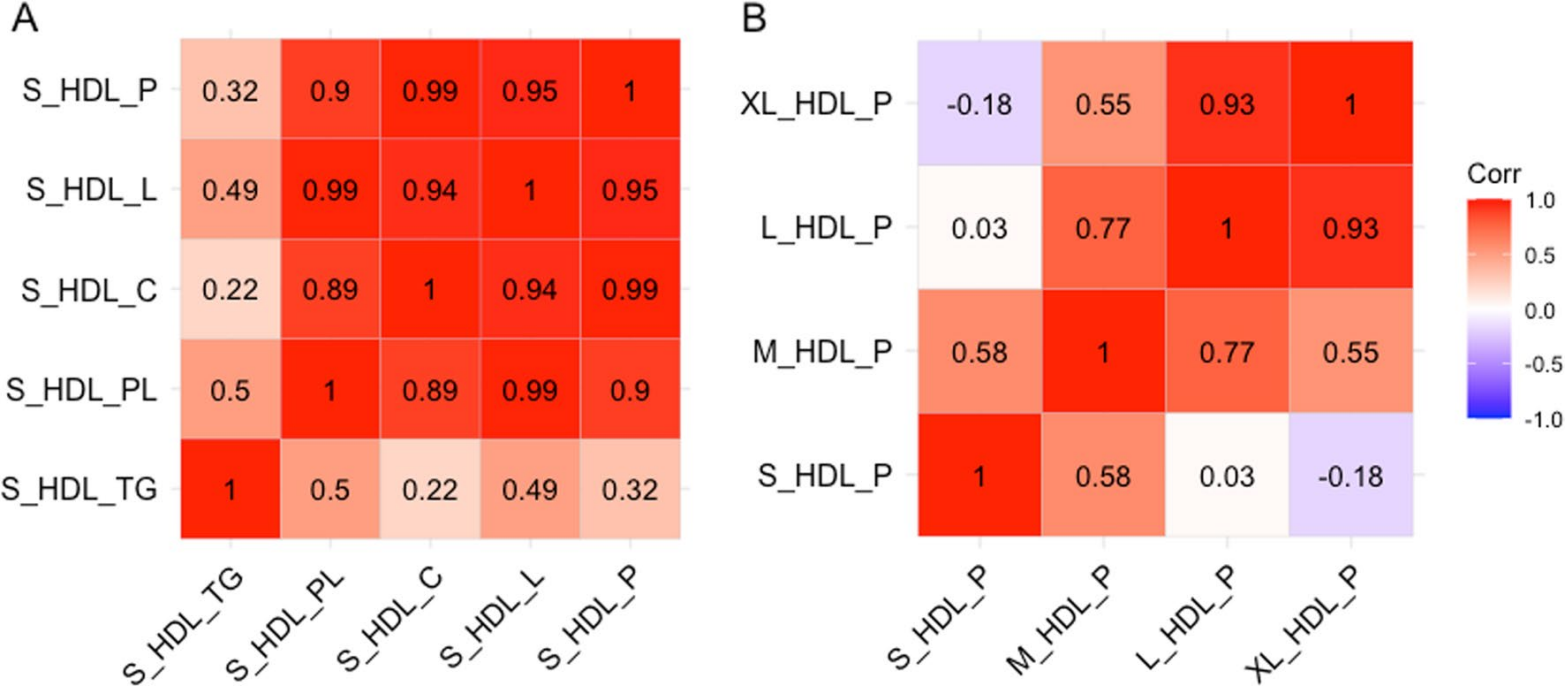
Correlation plots between **A** all small HDL lipid measures; showing high correlation between all measures, and **B** correlation of HDL particle count across different HDL sizes, showing the separation between small HDL cholesterol and other types of HDL cholesterol. Abbreviations: S_HDL_P = Concentration (particle count) of Small HDL particles; S_HDL_L = Total Lipids in small HDL; S_HDL_C = Cholesterol in Small HDL; S_HDL_PL = Phospholipids in Small HDL; S_HDL_TG = Triglycerides in Small HDL; XL_HDL_P = Concentration of Very Large HDL Particles; L_HDL_P = Concentration of Large HDL Particles; M_HDL_P = Concentration of Medium HDL Particles

Due to the extreme collinearity of HDL lipoprotein lipid measures, we performed initial analyses to identify clusters of correlated HDL measures. Figure 2 shows the dendrogram of these analyses, which show that correlation between HDL markers is largely driven by size, rather than content for lipid species outside triglycerides. This confirms the separation between small, medium, large and extra-large HDL clusters. Using a correlation cut of 0.2, seven separate clusters were identified, with small HDL markers clustering separately from other HDL subclasses. In line with this, the correlation between all lipid species measures of small HDL was very high (Pearson’s *R* > 0.7 for all, Fig. 3A) except small HDL TG, while the correlation between number of small HDL particles and particle counts for other HDL subclasses was low (Fig. 3B). Accordingly, we performed all subsequent analyses on the number of particles for each HDL subclass, as these results strongly correlated with other measures of particle content (e.g. concentration, number of cholesterol esters). [72]

### Repeat analyses

A total of 14,598 participants had HDL NMR metabolomic data measured at two time points (initial assessment, 2006–2008, second assessment 2012–2013). The median time between initial sample and repeat sample was 1574 days (~ 4.3 years; interquartile range 1353–1791 days). There was moderate stability of both small HDL and total HDL over this time point (Additional file 4: Figure S1), with the Pearson’s correlation between initial and repeat sample being 0.46 and 0.50, respectively.

### Observational analyses (analysis 1)

For each HDL subclass, we performed Cox regression for the number of particles and sepsis in turn, adjusted for age, sex, body mass index, C-reactive protein level, renal function, history of diabetes, history of liver disease, history of cancer, Townsend deprivation index, statin usage, smoking, and alcohol intake. For the linear models, HDL measures were scaled to have a mean of 0 and an SD of 1, to directly compare changes in exposure. When fitting each particle size individually, there was clear evidence that 1 SD higher levels of small HDL particles were associated with lower hazard of sepsis (HR 0.91; 95% CI 0.89–0.94, *p* = 2 × 10^–11^); whereas there was much weaker evidence for other particle sizes (all estimates in Additional file 1: Table S3).

The total particle count in all HDL particles per 1 SD higher values was also associated with lower hazard of sepsis (HR 0.93, 95% CI 0.90–0.96, *p* = 2 × 10^–6^), although this was weaker and more imprecise than the association with particle count in small HDL alone. To confirm that particle count in small HDL was driving the inverse association identified with particle count in total HDL, we ran analyses including particle count in small HDL and particle count in total HDL together in the same regression model. In these analyses, the inverse association with particle count in small HDL remained robust (HR 0.88; 95% CI 0.84–0.92, *p* = 9 × 10^–9^), while we identified no meaningful association with particle count in total HDL levels (HR 1.04; 95% CI 0.99–1.09, *p* = 0.12). For particle count in medium HDL, there was weak evidence of an inverse association in a linear model (HR 0.96; 95% CI 0.93–0.99, *p* = 0.01), but there was no evidence for particle count in other HDL particle size classes.

**Fig. 4:**
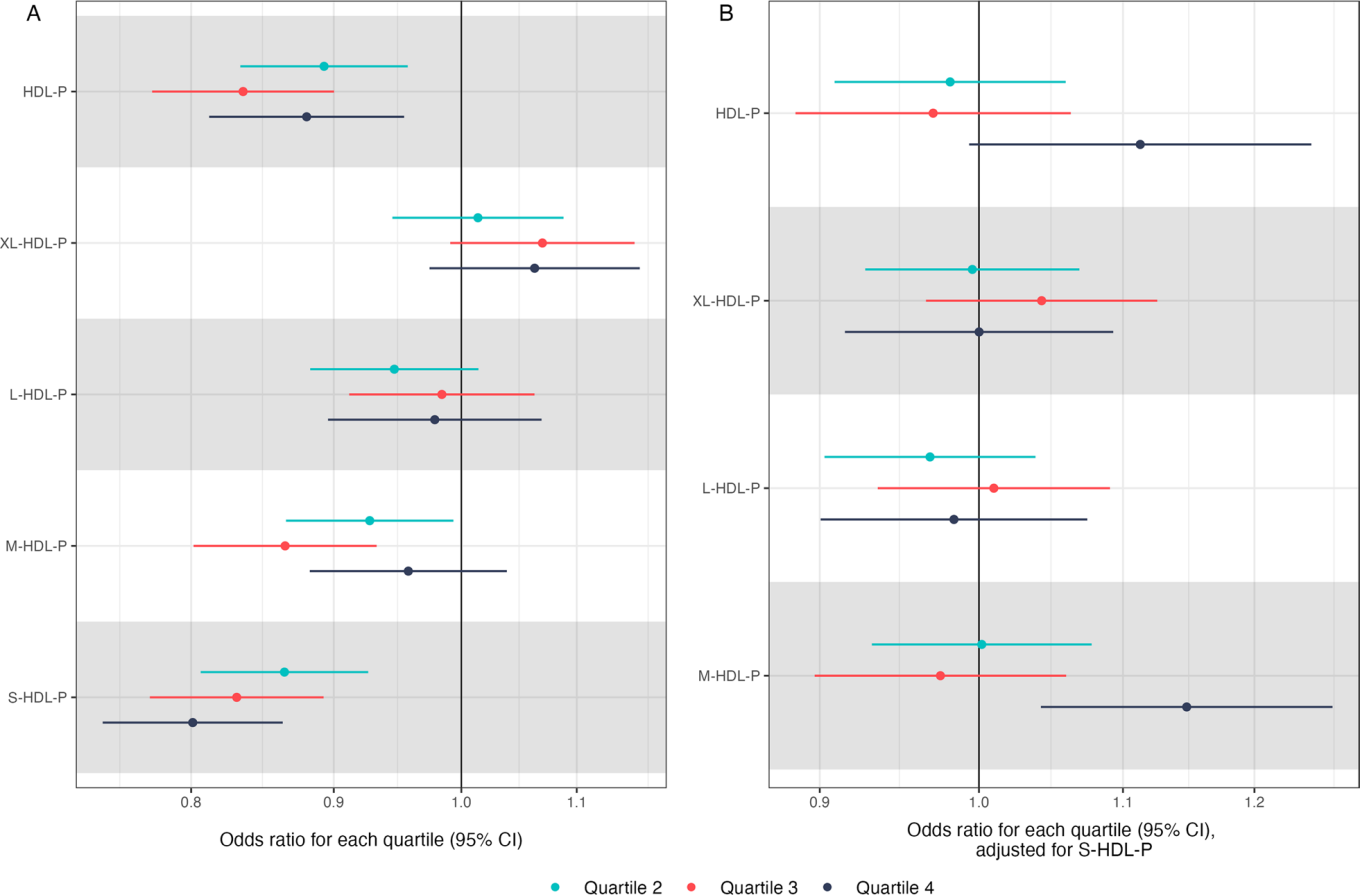
Associations between particle count of each HDL subclass size and the hazard of sepsis. All hazard ratios are compared with the reference (lower 25% of HDL particle counts, Quartile 1). Hazard ratios from adjusted Cox regression. Panel **A** shows these unadjusted for small HDL particle count; Panel **B** shows these adjusted for small HDL particle count. HDL-P = HDL particle count; XL-HDL-P = extra large HDL particle count; L-HDL-P = large HDL particle count; M-HDL-P = medium HDL particle count; S-HDL-P = small HDL particle count

When comparing quartiles, there was a monotonic decreasing hazard of sepsis with higher particle count in small HDL particles (Fig. 4A) but there appeared to be non-monotonicity in the association between the medium HDL particle count and the hazard of sepsis, with the strongest inverse association identified in Quartile 3 (HR 0.85; 95% CI 0.79–0.92; *p* = 4 × 10^–5^). When adding small HDL particle count to our models of other particle counts to explore whether small HDL particle count was driving associations identified in the particle count of other HDL particle sizes, given the correlation between particle counts across HDL subclass sizes. In these models, the association between particle counts and sepsis attenuated largely to the null, suggesting that other associations may reflect correlation with particle count in small HDL (Fig. 4B).

**Fig. 5:**
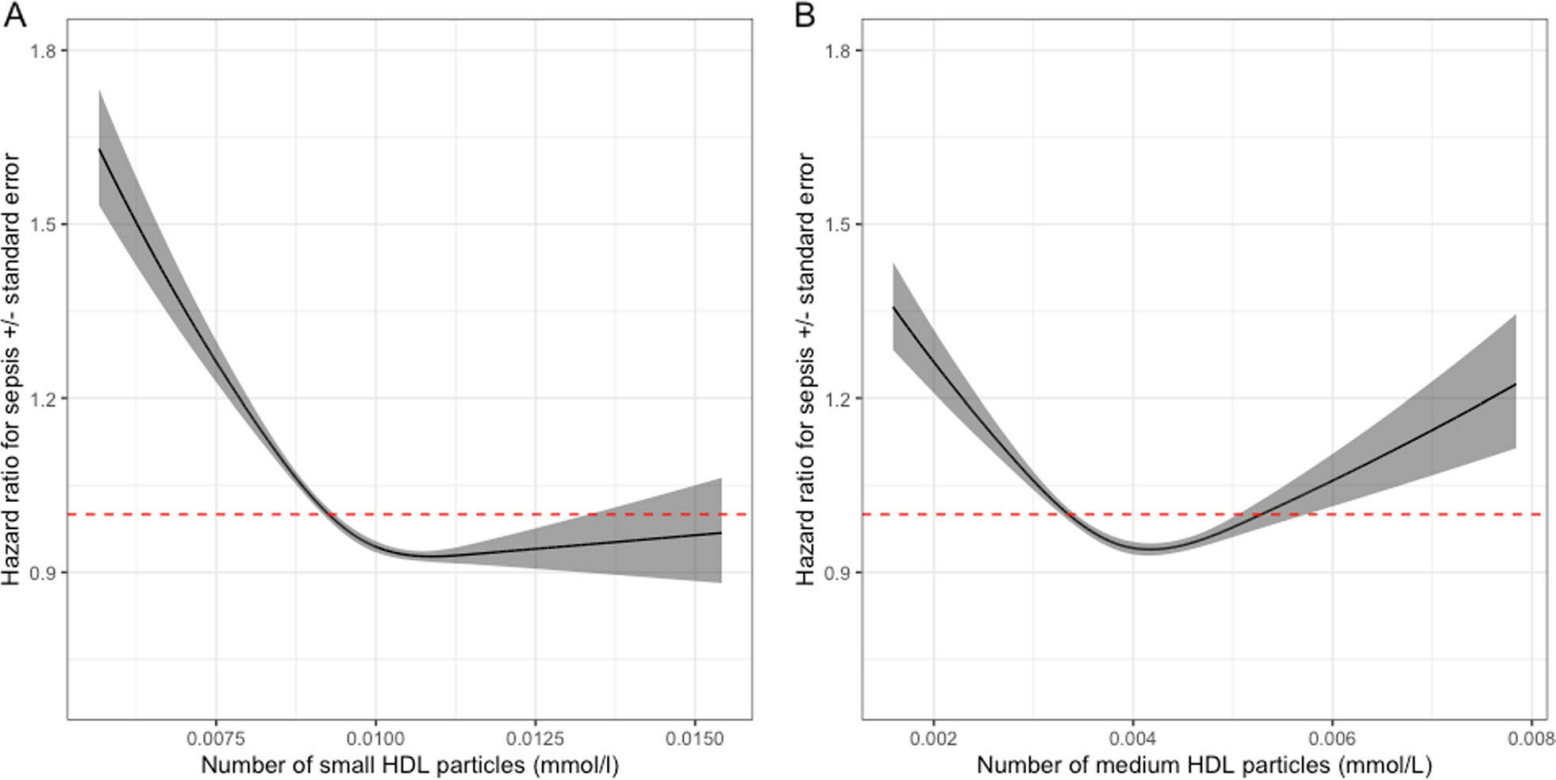
Restricted cubic spline modelling of the association between the hazard of sepsis and the number of small HDL particles (**A**) and medium HDL particles (**B**). Data from a model adjusted for all covariates listed in the methods. X-axis scale is per mmol/L. Data centred to remove points below the 0.1th centile and greater than the 99.9th centile

We then performed nonlinear modelling using restricted cubic splines. (Fig. 5, Additional file 4: Figure S2). In these spline models, the hazard ratio for incidence of sepsis was substantially higher at the extreme lower end of small HDL particles (Fig. 5A). The hazard ratio for being in the bottom 5% of small HDL particles vs. all other participants was 1.47 (95% CI 1.34–1.62), while for being in the bottom 1%, the hazard ratio was 1.72 (95% CI 1.45–2.04). There was clear visual evidence of nonlinearity, but no evidence of higher risk with higher particle numbers (e.g. a U-shaped relationship). For medium HDL particle number, there was more clear visual evidence of a U-shaped relationship in the association between medium HDL particle number, although the bulk of the hazard was still in those with lower medium HDL particle numbers (Fig. 5B). Evidence of non-monotonicity with other particle sizes was also somewhat evident in large HDL particle counts, but this was muted and much weaker than (Additional file 4: Figure S2) in the small and medium HDL particle counts.

In analyses adjusting for total triglycerides and LDL cholesterol, associations were less precisely estimated and slightly weaker, although the inverse association with increased particle count in small HDL remained (HR for lowest quartile of small HDL of 0.86; 95% CI 0.78–0.95, *p* = 0.001, Additional file 1: Table S4). The U-shaped association remained with particle count in medium HDL, with the lowest hazard in the middle two quartiles.

### Association between HDL particle size and sepsis-related mortality and critical care admission

**Fig. 6:**
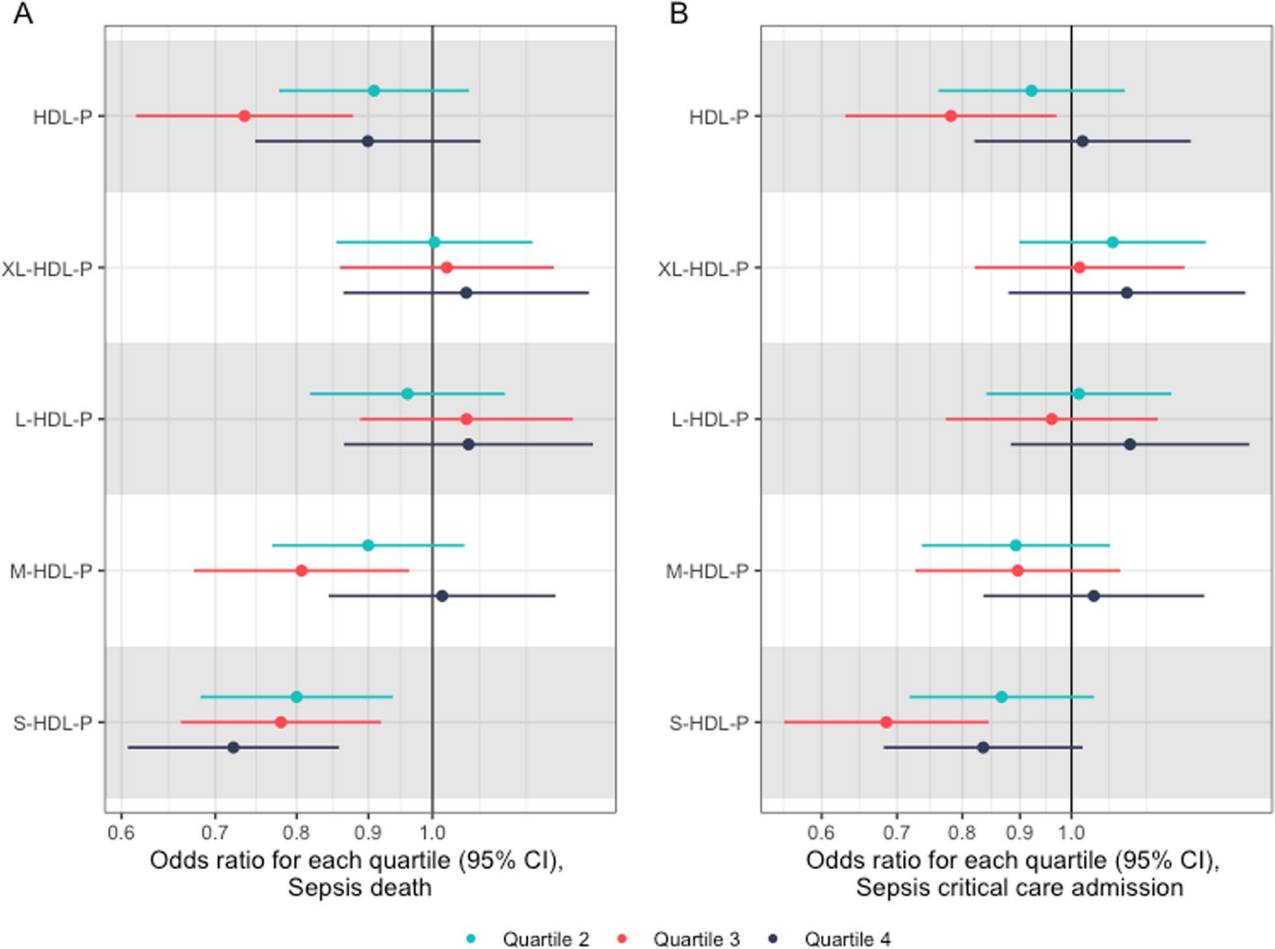
Associations between particle count of each HDL subclass size and the hazard of sepsis death (**A**) and sepsis critical care admission (**B**). All hazard ratios are compared with the reference (lower 25% of HDL particle counts, Quartile 1). Hazard ratios from adjusted Cox regression

In adjusted Cox regression models, a similar inverse association between higher levels of small HDL and lower sepsis-related death was identified (Fig. 6A). Given the smaller number of cases (1118 deaths), associations were less precise (HR 0.71; 95% CI 0.59–0.84, *p* = 0.0001, comparing Quartile 4; highest small HDL levels and Quartile 1; lowest small HDL levels). As with sepsis incidence, in restricted cubic spline models, the strongest evidence of increased hazard was in those with the lowest number of small HDL particles with a hazard ratio of 1.63 (95% CI 1.17–2.28) in the bottom 5%, and a HR of 2.50 (95% CI 1.46–4.28) in the bottom 1% (Fig. 6A). Particle count in Medium HDL again had a U-shaped association with sepsis-related death (Fig. 6B).

**Fig. 7:**
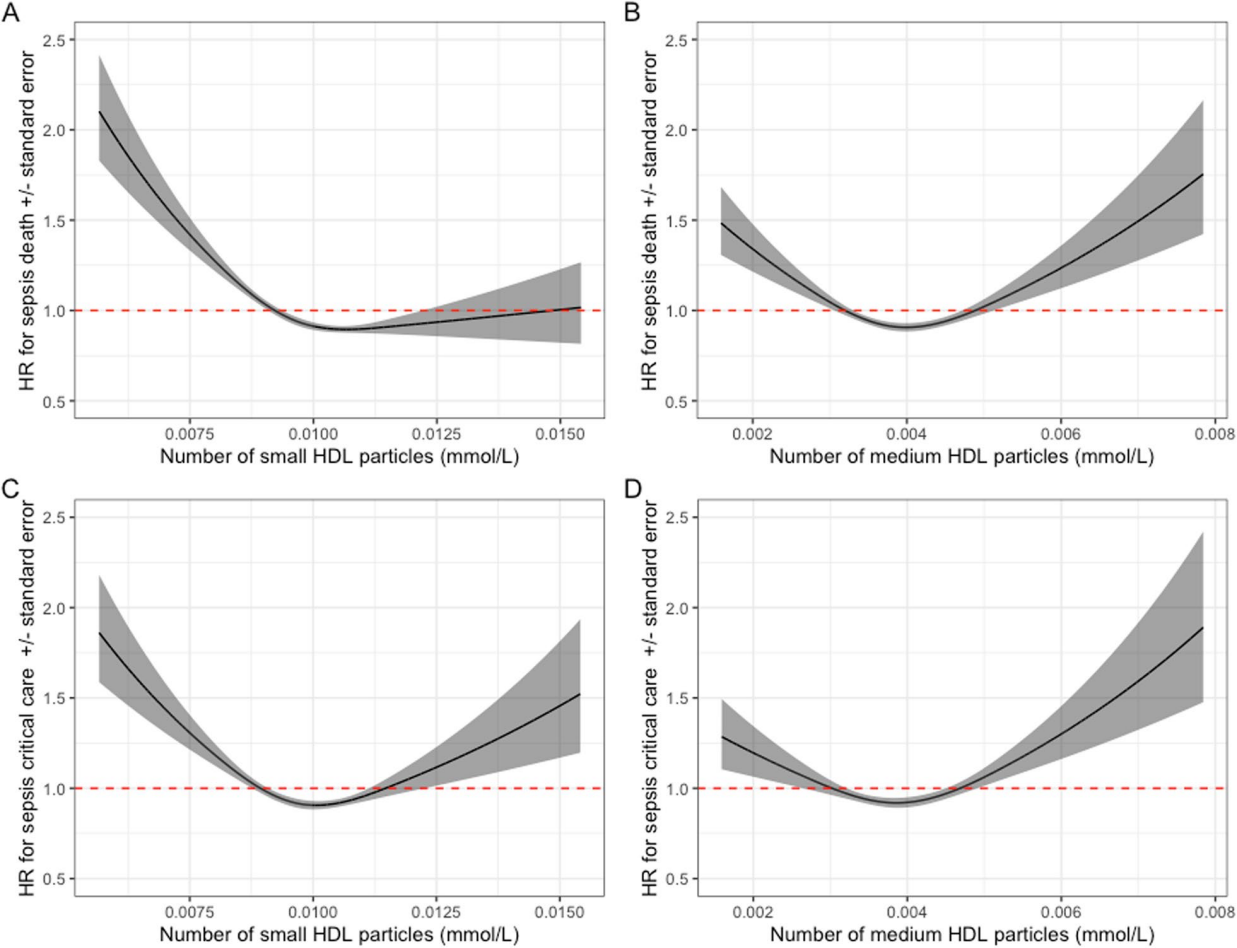
Hazard ratio of death and from sepsis with increasing number of small HDL particles (**A**) and medium HDL particles (**B**), and critical care admission with sepsis with small HDL (**C**) and medium HDL (**D**) particle counts. Estimates on the scale of hazard ratio, from adjusted Cox regression. Scale is per mmol/L. Graphs cut at the 0.1th centile and 99.9th centile to visualise the majority of the distribution

The evidence for a linear inverse association against critical care admission with sepsis was weaker (*p* for linear trend 0.06), although estimates were imprecise due to the small sample size. However, there was clear U-shaped relationship in both small and medium HDL particle counts and sepsis-related critical care admission when fitting restricted cubic spline models (Fig. 7).

Again, results for other HDL particle counts were attenuated to the null in models including small HDL particle count as a linear covariate (Additional file 1: Table S5).

### Does adjusting for CRP levels alter observed effect estimates?

As CRP—as marker of inflammation and IL-6 activity—is a potential confounder, and inclusion of CRP in models could lead to collider bias [73], we re-ran our primary analyses (on sepsis incidence, sepsis-related mortality, and sepsis-related death) with and without adjusting for CRP. We identified similar estimates for the association between small HDL and sepsis incidence and outcomes in the unadjusted models, with slightly larger effect sizes and narrower confidence intervals (e.g. OR for sepsis incidence in Quartile 4 of small HDL particle count 0.78 (95% CI, 0.72–0.84, *p* = 1.8 × 10^–11^) in models unadjusted for CRP, and 0.80 (95% CI 0.74–0.86, *p* = 4.9 × 10^–9^) in adjusted models. These results are fully reported in Additional file 1: Table S6.

### Is HDL causally associated with increased risk of sepsis? (Analysis 2)

Given the inverse linear association we identified was only observed for small HDL particle count, and there are no reliable nonlinear methods for MR available currently, we performed MR analyses on small HDL particle counts only. In the full UK Biobank cohort (*n* ~ 260,000) with small HDL measures, we performed a GWAS and identified 104 independent exposures. Variant details are reported in Additional file 1: Table S7. The median F statistic (a measure of the strength of the SNP-exposure association) was 2197 (lowest value 540). In sum, the variants explained 5.2% of the variation in small HDL levels.

Outcomes were measured in UK Biobank (sepsis incidence, sepsis death, and sepsis critical care admission) and FinnGen (sepsis incidence only). In inverse variance weighted meta-analysis, we identified no consistent predicted causal effect of particle count in small HDL on sepsis incidence, with a summary odds ratio of 1.00 (95% CI 0.95–1.10) when meta-analysing across UK Biobank and FinnGen. Results were similar in other meta-analytical approaches (weighted median, MR Egger, and when using MR-PRESSO, Additional file 1: Table S8).

We identified a modest predicted causal effect of small HDL particle count on sepsis-related critical care admission (OR 0.73; 95% CI 0.57–0.94). This effect should be taken with some degree of scepticism, given the number of outcomes tested, the weak and nonlinear observational association, and the play of chance. We report the other HDL subclass associations with each outcome in Additional file 1: Table S9. There was no consistent predicted causal effect of any other HDL particle count subclass on any sepsis outcome, in line with the observational data.

Finally, to explore the phenotypic effects of the genetic variation associated with small HDL particle count, we generated a PRS for small HDL particle count and whether this associated with counfounders. We then split participants based on their quartile of polygenic risk score and explored the demographics, which showed some evidence that those with different levels of the PRS had different levels of covariates (Additional file 1: Table S10).

### The bidirectional association between IL-6 and CRP signalling and the effect on HDL subclasses (analysis 3)

**Fig. 8:**
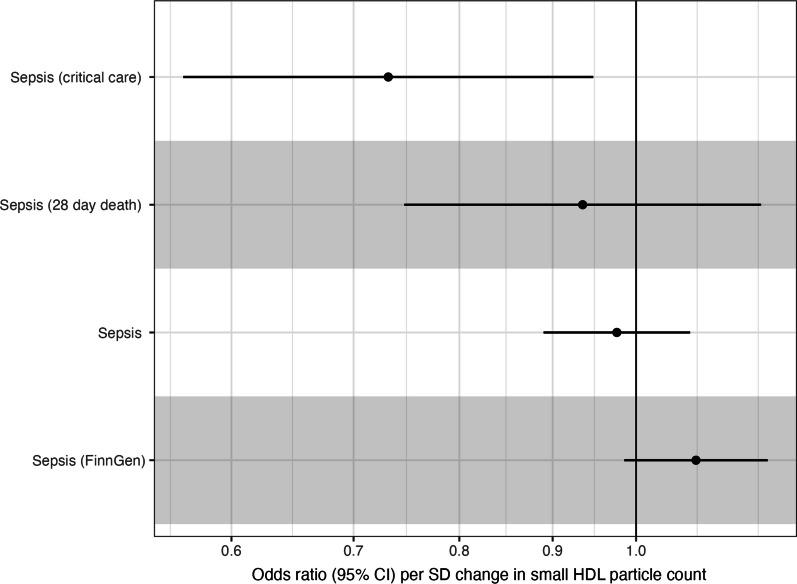
Inverse variance weighted meta-analytic MR results of the effect of each HDL particle count and subclass on the odds of various sepsis outcomes. Scale is per one SD increase in small HDL particle counts. The top three outcomes were measured in UK Biobank and the bottom outcome (labelled FinnGen) was measured in FinnGen

To explore potential reasons for the strong observational association and (largely) null genetic association, we performed further MR analyses to explore whether IL-6 signalling (which is known to alter the risk of infection [73]) could alter particle count of small HDL leading to our observed results. To do this, we performed bidirectional MR, first looking at whether IL-6 levels (modelled using *cis IL6R* variants) and CRP levels (modelled using *cis CRP* variants) are causally associated with HDL subclass particle counts. We then subsequently performed the “reverse” analysis, identifying whether changes in HDL subclasses altered IL-6 and CRP levels (Fig. 8).

**Fig. 9:**
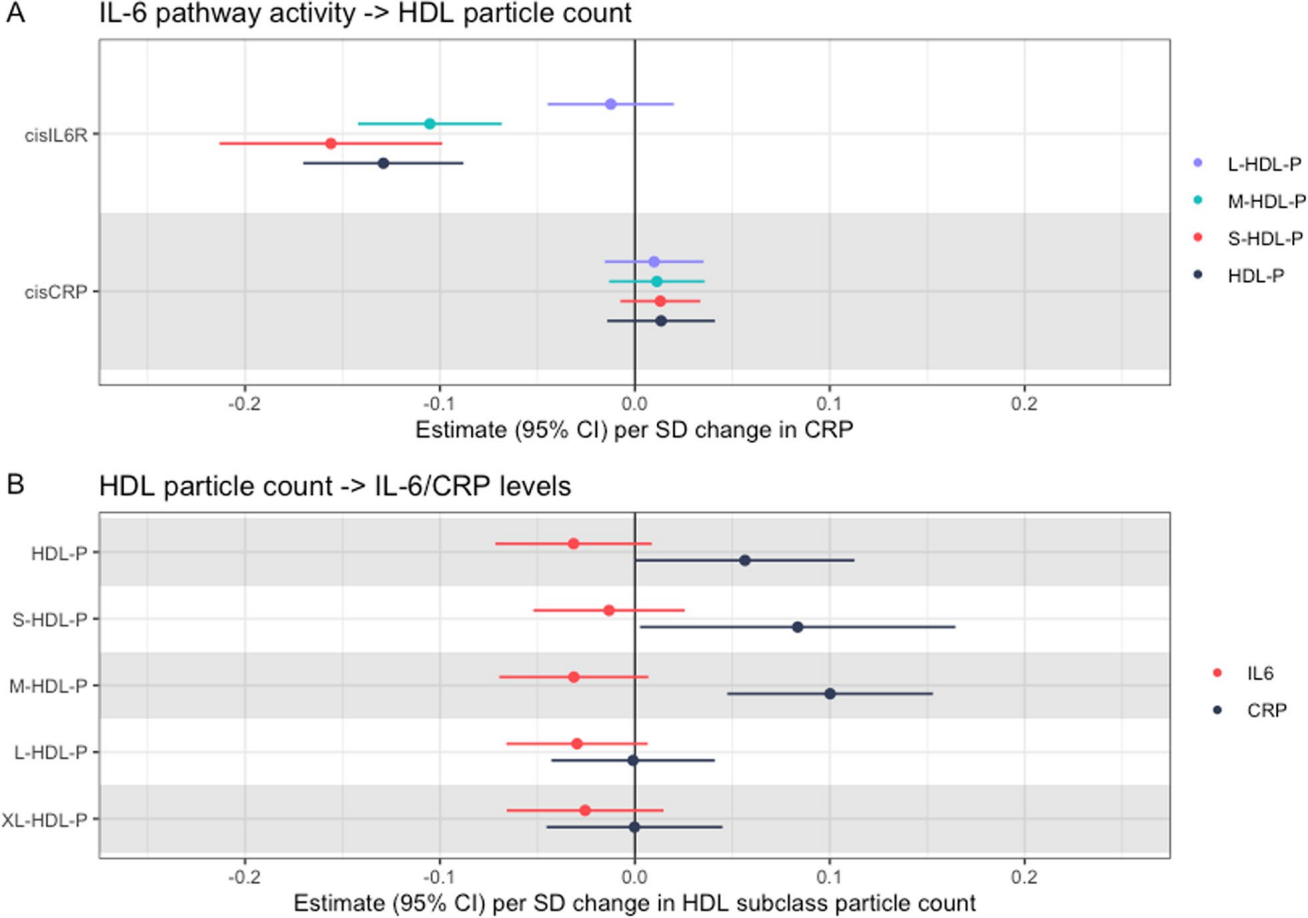
The effect of IL-6 and CRP pathway activity on HDL subclass particle counts (**A**), and the effect of HDL particle counts on IL-6 and CRP levels (**B**) Estimates were generated using cisIL6R variants to proxy the effect of increased IL-6 pathway activity and using cisCRP variants to proxy the effect of increased CRP protein. Estimates are on the scale of 1 SD change in each exposure

In our analysis on the effect of IL-6 and CRP signalling on HDL subclasses, we saw a large predicted causal effect of increased IL-6 signalling and reduced particle count in small HDL (Fig. 9A), beta on small HDL particle count − 0.16, 95% CI − 0.10 to − 0.21 per natural log change in SD-scaled CRP, *p* = 9 × 10^–8^). We saw a similar effect on particle count in total HDL, with increased IL-6 signalling predicted to reduce the particle count in total HDL (beta − 0.13, 95% CI − 0.09 to 0.17, *p* = 7 × 10^–10^), and with medium HDL (beta − 0.11, 95% CI − 0.14 to − 0.07, *p* = 2 × 10^–8^). We saw no effect on larger HDL particle sizes. Using our *cis* CRP variants—that represent the effect driven by alterations in CRP protein levels—we saw no predicted causal effect on any HDL subclass.

We then analysed the effect of increasing HDL subclasses on IL-6 and CRP levels (Fig. 9B). We identified no strong evidence of a causal effect of increased HDL on IL-6 levels, although confidence intervals did not preclude a biologically relevant effect size, and all estimates were below zero. We identified a possible positive effect of small and medium HDL on CRP levels although estimates were imprecise, beta for small HDL 0.08 (95% CI 0.003–0.16, *p* = 0.04; beta for medium HDL 0.1; 95% CI 0.05–0.15, *p* = 0.001).

To summarise, we identified robust evidence that increased IL-6 pathway activity is predicted to decrease levels of total, medium, and small HDL particle counts. In contrast, increased levels of CRP itself appeared to have no predicted effect. In the other direction, increased levels of medium HDL and possibly small HDL particle count increased CRP levels but had no effect on IL-6 levels.

### Effect of statin usage on small HDL exposures

Due to the potential effect of statins on HDL measures, we performed GWAS of small HDL measures in statin users and non-statin users in order to compare estimates. As stratifying on statin use might generate a collider bias (as statin use is driven by cholesterol measurements), we additionally performed a GWAS of small HDL in UK Biobank participants under 50 at the time of sampling (*n* = 23,532, where statin use was rare (4%, *n* = 994). We then performed LD score regression on these three datasets. As would be expected given the minimal effect of statin usage on small HDL (Additional file 4: Figure S3), the genetic correlation between all three small HDL GWAS was very high: (rg between statin users and non-statin users 1.00, rg between non-statin users and participants under 50 0.99; rg between statin users and participants under 50 0.92), suggesting limited bias due to statin usage.

### Block jacknife resampling

As biased estimates can potentially be produced with overlapping exposure and outcome GWAS, we performed block jacknife resampling [72] to estimate the effect of small HDL particle count on sepsis incidence. This had little effect on estimates, so we did not perform this for other analyses. These results are reported in an Additional file 5.

## Discussion

In this study, we used a large (*n* ~ 260,000) cohort of participants with NMR-based data on HDL subclasses and identified a robust observational association between low number of small HDL particles and increased hazard of sepsis, sepsis-related death, and sepsis-related critical care admission. MR data did not generally support a causal effect of small HDL particle count per se on sepsis. However, we did identify a robust causal association between increased IL-6 pathway activity and reduced (small) HDL particle count. We did not identify strong evidence of a reverse effect, that is increased (small) HDL levels leading to reduced IL-6 signalling (in contrast to some experimental data [74]). In fact, there was weak evidence of a CRP increasing effect on small HDL particle count, although the magnitude of this was smaller than the IL-6 signalling to HDL association and there was imprecision around estimates. In concert with trial, registry, and MR data supporting the causal effect of altering IL-6 pathway activity on infection [75], these data support the view that observed HDL associations with sepsis are—in part—downstream of IL-6 signalling.

Indeed right at the start of clinical trials of interferon treatment, a persistent drop in HDL levels during treatment was repeatedly reported and Cantell in 1980 was the first to suggest that infections elicit a drop in HDL via the induction of an inflammatory interferon response [75]. In this regard, we note a wide range of infections cause a drop in HDL and thus the response is not pathogen specific and suggestive for a common host-derived mediator. Interferons amongst other pro-inflammatory cytokines, such as Interleukin-1 beta (IL-1b) and IL-6, activate the acute phase response in the liver with IL-6 exhibiting the strongest effect. The acute phase response is by definition those serum proteins that increase or decrease their circulatory concentrations by 25% or more, representing positive and negative reactants, respectively, a response that may occur not only acutely but also chronically [76]. This raises the notion for shifts in the baseline immune inflammatory balance affecting acute phase reactants and the metabolic development of HDL particles. Supporting, this, serum amyloid A (SAA), an acute phase protein whose production is controlled by IL-6 is known to modulate HDL function and to displace apolipoprotein-A from HDL molecules [77].

It is important to note that IL-6 signalling did not account for all of the observed risk of sepsis with reduced small HDL particle count in either our observational analysis (adjusting for CRP) or our genetic analyses. As HDL particle count does not appear to be causal for sepsis, this suggests other unmeasured causal confounder(s) are present. These confounders may even be other aspects of small HDL biology that are co-incident with particle count. NMR measures only reflect particle count, and may well not reflect other characteristics or functional aspects of HDL metabolism that are relevant for protection from infection [78, 79]. These data should not, therefore, be interpreted as suggesting (small) HDL has no relevant role in infection, but simply that small HDL particle count, as determined by NMR, is not causal for sepsis.

It is also important to understand the relative complexity of interpreting an instrument for a (small) lifetime change in an exposure and sepsis which represents a severe phenotype of infection. Sepsis is a life-threatening organ dysfunction caused by a dysregulated host response to infection, and so associations with sepsis can represent either an increased risk of susceptibility to symptomatic presentation through reduced defensive mechanisms (for example, in those with immunosuppression), or increased rate of sepsis development within infection by unbalanced host–pathogen interactions (e.g. strains of *Streptococcus pyogenes* that carry toxins) [80]. It is possible, and perhaps likely, that exposures may have differing effects on each of these stages, making interpretation of genetic estimates complex. Additionally, observational data suggest higher rates of infection in patients with organ transplant, but lower rates of severe infection [81–83].

In summary, this study confirms recent data that suggest that the longstanding relationship between HDL [82] and sepsis is likely limited to the small (and perhaps medium) HDL component of HDL. Particle counts of small HDL appear to be largely downstream of IL-6 signalling, which likely confound observed estimates between HDL measures and infection. There is weak evidence of an effect of small HDL on critical care admission with sepsis, which should be explored in other cohorts.

### Strengths and limitations

The major strength of this study is the scale and quality of the dataset, with high quality, prospectively collected, linked electronic health record data, genetic data, medication data, and detailed lipoprotein subclasses data based on NMR spectroscopy available for a large number of participants. However, like all epidemiological studies, it has a number of limitations.

Firstly, UK Biobank is not a representative cohort, with multiple studies identifying the population to be healthier than the UK population on many metrics [84]. The bias induced on selection of this cohort therefore has the potential to affect estimates, although our results were reassuringly similar to estimates generated from an independent cohort [84].

Secondly, the ascertainment of sepsis cases remains limited to hospital records. Although these are widely used for epidemiological studies, coding is recognised to be imperfect and there is limited information on, e.g. type of pathogen. Although our MR data did not show a causal effect of (small) HDL on sepsis, the presence of > 100 genome wide significant “independent” SNPs for small HDL particle count underscore the polygenicity and multiple metabolic pathways in play.  Further, the effect of IL-6 signalling on HDL suggests that HDL is involved in the innate immune response. Future research focussed on dissection these pathways and functional forms of HDL will be helpful for further understanding whether any of these identified observational associations are causal, or whether drugs targeting specific HDL-pathway biology (e.g. CETP inhibitors) may have a protective role [41, 85, 86].

We also have to be conscious that our NMR measures were taken in a healthy cohort at steady state. Although MR estimates generated from steady state have been shown to be relevant in acute infection (e.g. the successful randomised trials of baricitinib for critical COVID-19 were based on MR evidence generated from *cis *variants known to alter *TYK2* expression in patients at steady state [85, 86]), we should expect the levels of these particles and subsequent genetic associations are likely to differ in acute infection.

Finally, although the Nightingale platform used in this study has been widely used, some groups have raised concerns about the accuracy and comparison with other methodologies (particularly around LDL measures) [87]. These criticisms have in turn been questioned, and the platform has shown strong predictive ability for multiple diseases in multiple cohorts [41, 88, 89]. Additionally, we present data provided by Nightingale health comparing HPLC measures with that measured by the Nightingale platform (Additional file 4: Figure S4). These data show good correlation (Pearson’s *R* 0.82–0.97) for each HDL subclass.

### Comparison with previous literature

To our knowledge, only one previous study has examined the associations between HDL subclasses and subsequent infectious disease outcomes [29]. That study, performed in 30,195 participants of the Copenhagen General Population Study, focussed on all infectious disease outcomes, not only sepsis. In that study, small and medium HDL particles were combined. In line with our study, they identified an association with increased numbers of small and medium HDL particles and protection from sepsis, with effect sizes in line with our data. However, we add to this finding, (a) by identifying the nearly linear association with particle concentration of small HDL, but nonlinear association with particle concentration of medium/large HDL, (b) providing more precision around estimates, (c) using genetic data, which did not support a causal relationship between total HDL and small HDL on risk of sepsis, and (d) adding further analyses on causal relationship with IL-6 signalling explaining the relationship between low number of small HDL particles and increased risk of sepsis.

One previous MR analysis identified a modest protective effect of total HDL cholesterol (not particle count) on infection outcomes in an analysis in UK Biobank, although estimates were imprecise [20]. The difference between our results may reflect the differing traits measured, which are incompletely correlated, and may represent differing biology. Additionally, as follow-up periods have increased (their follow-up ended 2016; ours 2021) our precision for outcome measures is greater, and the closer to null result may better reflect the true estimate [90].

## Conclusions

In a large, prospective cohort study, lower particle counts of small HDL, but not other HDL sizes, were robustly associated with increased hazard of sepsis hospitalisation, sepsis-related mortality, and sepsis-related critical care admission. However, genetic analyses did not reveal strong evidence for causation for small HDL per se, and suggested inflammation via IL-6 signalling as a potential explanatory variable.

### Supplementary Information

**Additional file 1.** ST8: Alternative meta-analytic strategies, and alternative MR methods for analysing the genetic data.

**Additional file 2.** GWAS methodology.

**Additional file 3.** STROBE-MR checklist of recommended items to address in reports of Mendelian randomization studies.

**Additional file 4.** Supplementary Figures.

**Additional file 5. Figure 1:** Estimates of the effect of small HDL particle count on sepsis incidence using block jacknife resampling in UK Biobank. Estimates on the scale of one SD change in small HDL particle count and are generated by inverse variance weighted MR.

## Data Availability

Individual level data are available via access to the UK Biobank. For the genetic analysis, we provide harmonised summary statistics at https://github.com/gushamilton/small_hdl_sepsis/tree/main for ease of replication. Our UK Biobank GWAS on HDL subclasses performed as part of this study is available at https://figshare.com/articles/dataset/HDL_GWAS/24024816.
