# Supplementary material for: Low levels of small HDL particles predict but do not influence risk of sepsis
Source: Crit Care. 2023 Oct 9;27:389. doi: 10.1186/s13054-023-04589-1 (PMC10563213; doi:10.1186/s13054-023-04589-1)
Supplement: Supplementary file 2 — Additional file 2. GWAS methodology. [file 13054_2023_4589_MOESM2_ESM.docx]

*Genetic data*

GWAS were performed on imputed genetic array data held within the MRC-IEU at the University of Bristol. The sample was limited to those of European ancestry. Details on imputation approach quality control, definition of ancestry, and sample and variant level exclusions are available elsewhere.^42^

*GWAS methodology*

We performed a GWAS using regenie 2.6.2, on the imputed quality controlled genetic data held at the MRC-IEU;^42,46^ regenie effectively controls for case-control imbalance and population structure. GWAS was performed adjusting for age, sex, genetic chip, and recruitment centre. For our exposure GWAS, we performed a GWAS on the measured small HDL particle count numbers after removal of technical variation and normalization. Therefore, betas are on the scale of 1 SD change in small HDL. For our outcome GWAS on sepsis, we defined outcomes as in our observational analyses, and performed a case-control GWAS, leading to log-odds ratios for each estimate. We also performed GWAS of particle count of other particle sizes (medium, large, extra-large) and of total HDL particle count without small HDL for use as outcomes in our IL-6 analysis which is described below. The methodology described is the same.

*Avoiding sample overlap*

Although summary statistics from GWAS of both sepsis^40,41^ and HDL subclass measures^43^ are available already, the largest samples (by an order of magnitude) is in UK Biobank, leading to sample overlap. Sample overlap in the exposure and outcome datasets leads to “winners curse”, biasing estimates.^37,44^ In order to maximize sample size while reducing overfitting, we therefore split UK Biobank into multiple blocks using a block jacknife approach. Firstly, we performed a GWAS on all ~ 260,000 participants with small HDL particle count numbers and genetic data on recruitment to UK Biobank and used this to generate instruments for small HDL. We then measured outcomes (by performing a GWAS for each outcome) in the ~ 210,000 participants who had no small HDL measures. As there is no sample overlap between these groups, this represents traditional two sample MR.

To measure outcomes in the remaining ~ 260,000 participants we used a block resampling approach.^45^ To do this we split this group into ten further samples, each with around 26,000 participants. We measured the outcome in each block independently and used a GWAS for the exposure performed in the other blocks to generate instruments without sample overlap. For example, we combined groups 1-9 and performed a GWAS of small HDL measures (n ~ 234,000). We then generated instruments (described below) in this block and used these instruments on outcomes we had measured in block 10 (n ~ 26,000). We then repeated this for all other blocks. In this way, ten estimates are generated (one for each block). These estimates were then combined, along with the estimate in the 210,000 UK Biobank participants without small HDL measures, to generate a summary MR estimate, using fixed effects meta-analysis. This approach has been shown to effectively reduce bias from sample overlap.^45^ For ease, we present a summary MR estimate for the whole of the UK Biobank in the results, but present individual block level estimates in the supplementary results. The results of the summary MR estimate was however similar to when sample overlap was ignored, that is, doing a GWAS on all ~ 260,000 participants for HDL particle number followed by outcomes GWAS on all ~ 260,000+210,000 = 470,000 participants, suggesting this bias was minimal.
