# Supplementary material for: Low levels of small HDL particles predict but do not influence risk of sepsis
Source: Crit Care. 2023 Oct 9;27:389. doi: 10.1186/s13054-023-04589-1 (PMC10563213; doi:10.1186/s13054-023-04589-1)
Supplement: Supplementary file 4 — Additional file 4. Supplementary Figures. [file 13054_2023_4589_MOESM4_ESM.docx]

**Supplementary Figure S1**: Correlation between HDL measures for a) small HDL particle count, and b) total HDL particle count taken at recruitment to UK Biobank and at reassessment (n= 14,589).


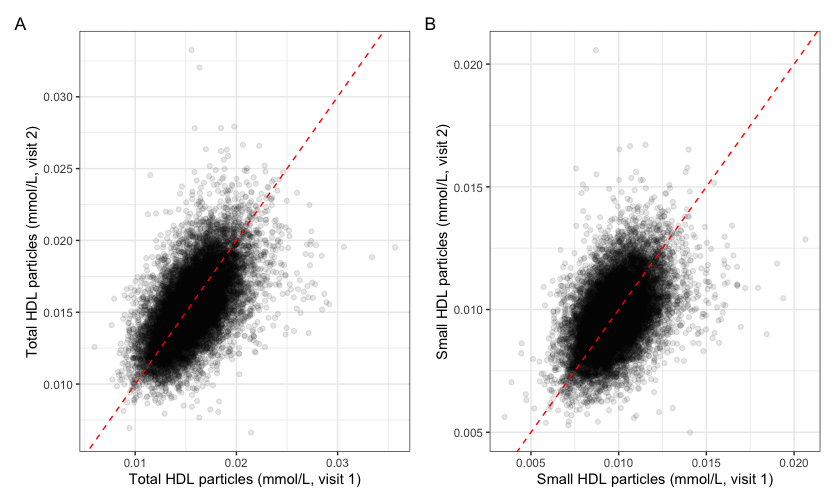


**Supplementary Figure S2**: Association between large (A) and extra large (B) HDL measure and the hazard of sepsis. Estimates from a restricted cubic spline model, adjusted for all the covariates listed in the methods. These are presented on the scale of mmol/L.

**
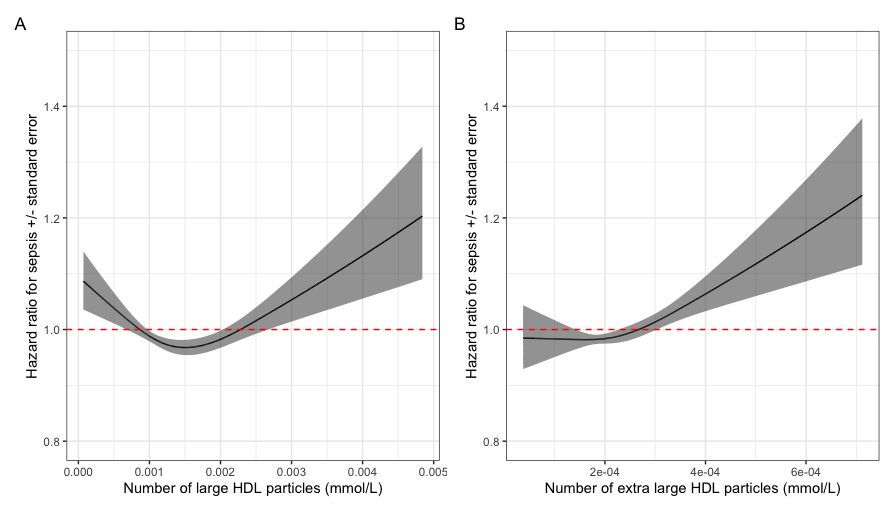
**

**Supplementary Figure S3**: Association between statin use and Small HDL particle count.


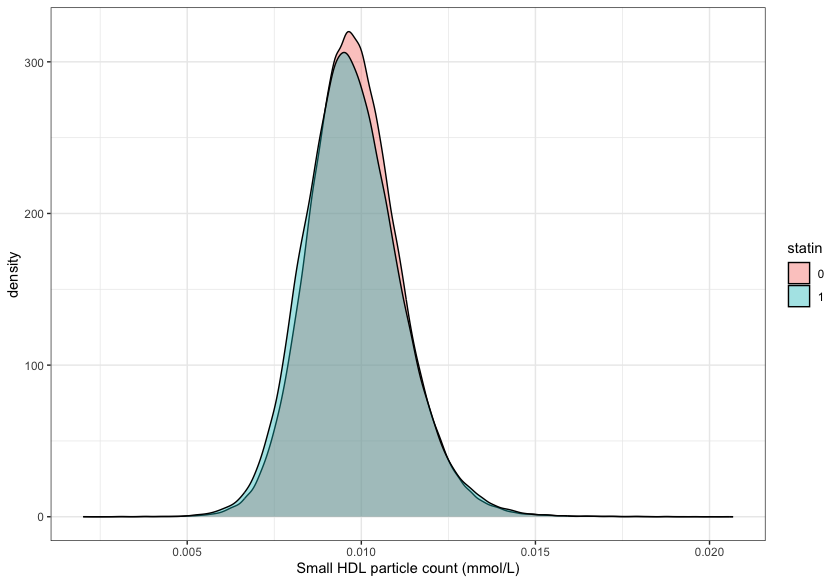


**Supplementary Figure S4:**  Scatter plots of the Nightingale Health NMR data for HDL subclass particle numbers when calibrated against gel permeation high-performance liquid chromatography (GP HPLC^1^). The NMR-based values were derived from spectral data calibrated to the HPLC measurements in around 300 samples.^2^ The results shown are from five-fold cross-validation. The average particle diameter of the HDL subclasses calibrated via GC HPLC are as follows: extra large 14.3 nm, large 12.1 nm, medium 10.9 nm, and small 8.7 nm. R denotes Pearson correlation coefficients.

**
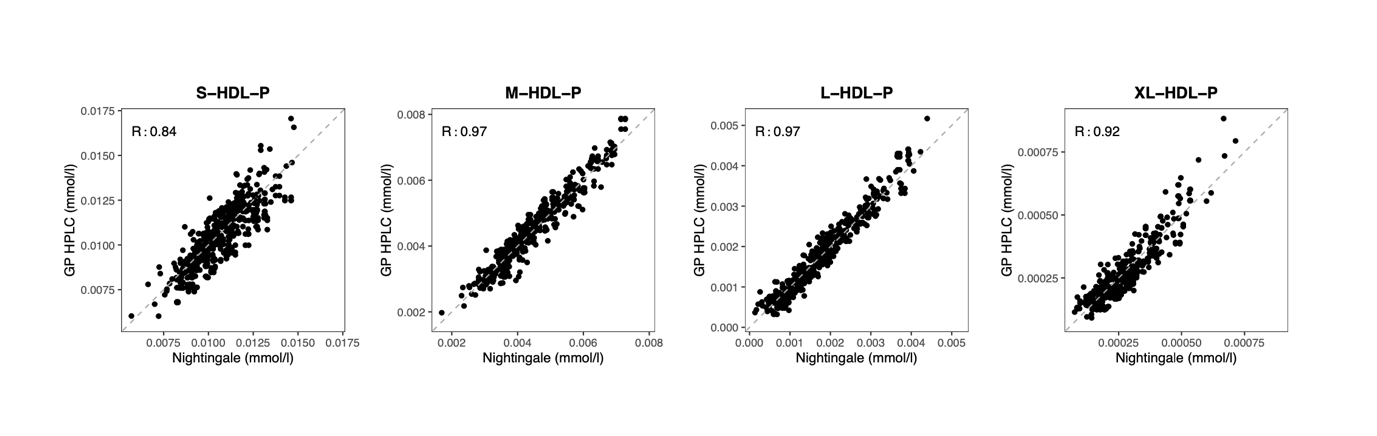
**

1. Okazaki M, Yamashita S. Recent Advances in Analytical Methods on Lipoprotein Subclasses: Calculation of Particle Numbers from Lipid Levels by Gel Permeation HPLC Using “Spherical Particle Model.” J Oleo Sci [Internet] 2016;65(4):265–82. Available from: http://dx.doi.org/10.5650/jos.ess16020

2. Würtz P, Soininen P. Reply to: “Methodological issues regarding: ‘A third of nonfasting plasma cholesterol is in remnant lipoproteins: Lipoprotein subclass profiling in 9293 individuals’” [Internet]. Atherosclerosis. 2020;302:59–61. Available from: http://dx.doi.org/10.1016/j.atherosclerosis.2020.03.028
