# Supplementary material for: Low levels of small HDL particles predict but do not influence risk of sepsis
Source: Crit Care. 2023 Oct 9;27:389. doi: 10.1186/s13054-023-04589-1 (PMC10563213; doi:10.1186/s13054-023-04589-1)
Supplement: Supplementary file 5 — Additional file 5. Figure 1: Estimates of the effect of small HDL particle count on sepsis incidence using block jacknife resampling in UK Biobank. Estimates on the scale of one SD change in small HDL particle count and are generated by inverse variance weighted MR. [file 13054_2023_4589_MOESM5_ESM.docx]

Supplement 1:

Block jacknife resampling was performed for the association between small HDL particle count and sepsis. As this is computationally expensive and potentially wasteful^1^, requiring 22 GWAS to be performed for each exposure-outcome association, and simulations do not suggest strongly biased estimates with highly powered exposures^2^, we only tested small HDL particle count on sepsis incidence. In this analysis, we split the participants of UK Biobank into overlapping blocks (see methods for details). Each block had approximately 233,846 participants in it. We generated instruments for small HDL particle count within each block, and performed MR on the ‘hold-out’ proportion of the sample that was not overlapped with this block. Full details of this method are described elsewhere.^3^ As expected, given the sample overlap between each block (90%), exposures were similar. We identified between 90 and 104 independent SNPs in each block to perform MR. 11 separate MR analyses were then performed, for the 10 overlapping blocks and their corresponding hold out sample and then using all participants with HDL measures on the sample of UK Biobank without any HDL measures. Results are shown in the figure **Supplement S1 Figure 1** below. The summary fixed effects estimate was an odds ratio of 0.952 (95% CI 0.866 - 1.05), which was very similar to the estimate generated without accounting for sample overlap (OR of 0.976 (95% CI 0.89 - 1.07). We therefore chose to not perform this for other analyses in this paper.

**Supplement S1 Figure 1:** Estimates of the effect of small HDL particle count on sepsis incidence using block jacknife resampling in UK Biobank. Estimates on the scale of one SD change in small HDL particle count and are generated by inverse variance weighted MR.


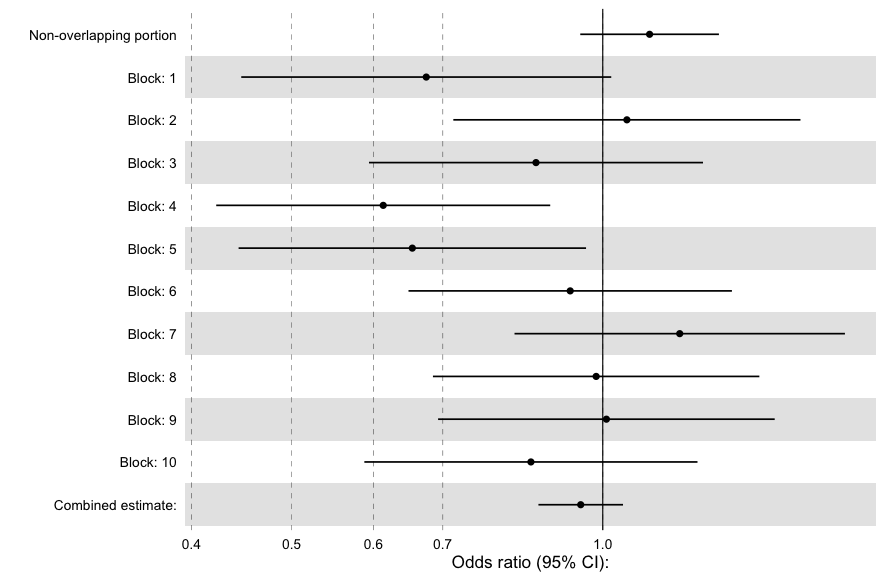


1. Lannelongue L, Grealey J, Bateman A, Inouye M. Ten simple rules to make your computing more environmentally sustainable. PLoS Comput Biol [Internet] 2021;17(9):e1009324. Available from: http://dx.doi.org/10.1371/journal.pcbi.1009324

2. Sadreev II, Elsworth BL, Mitchell RE, et al. Navigating sample overlap, winner’s curse and weak instrument bias in Mendelian randomization studies using the UK Biobank [Internet]. medRxiv. 2021 [cited 2022 Apr 28];2021.06.28.21259622. Available from: https://www.medrxiv.org/content/10.1101/2021.06.28.21259622v1.abstract

3. Fang S, Hemani G, Richardson TG, Gaunt TR, Davey Smith G. Evaluating and implementing block jackknife resampling Mendelian randomization to mitigate bias induced by overlapping samples. Hum Mol Genet [Internet] 2023;32(2):192–203. Available from: http://dx.doi.org/10.1093/hmg/ddac186
